# 

**Published:** 1898-09

**Authors:** 


### BOOKS RECEIVED.

A Guide to the Clinical Examination of the Blood for Diagnostic Purposes. By Richard C. Cabot, M. D. With colored plates and engravings. Third revised edition. Octavo, pp. 462. Price, $3.25. New York: William Wood & Co. 1898.

A Text-book upon the Pathogenic Bacteria for Students of Medicine and Physicians. By Joseph McFarland, M. D., Professor of Pathology in the Medico-Chirurgical College, Philadelphia, etc., etc. Octavo, pp. 497. Price, $2.50 net. With 134 illustrations. Second edition, revised and enlarged. Philadelphia: W. B. Saunders. 1898.

Lectures on Tumors. By John B. Hamilton, M. D., LL. D., Professor of Surgery, Rush Medical College and Chicago Polyclinic; Surgeon to Presbyterian Hospital, etc., etc. Third edition. Illustrated. Price, $1.25 net. Philadelphia: P. Blakiston's Son & Co. 1898.

Transactions of the Medical Association of Central New York. Thirtieth annual meeting held at Buffalo, N. Y., October 19, 1897. Charles A. Van der Beek, M. D., Secretary. Volume IV. Buffalo Medical Journal Print. 1898.

Proceedings of the Tenth Annual Session of the Association of American Anatomists. Held at Cornell University, Ithaca, December 28–30, 1897. D. S. Lamb, M. D., Secretary.

A Manual of Surgery. For Students and Practitioners. By William Rose, M. B., B. S. Lond., F. R. C. S., and Albert Carless, M. S. Lond., F. R. C. S. One volume, 8vo, of 1,162 pages, very profusely illustrated by wood engravings. Extra muslin, $5.00 net; leather, $5.75 net. New York: William Wood & Co. 1898.

Handbook for the Hospital Corps of the U. S. Army and State Military Forces. By Charles Smart, Deputy Surgeon-General U. S. A. Approved by the Surgeon-General of the Army. Second revised edition. 12mo, 358 pages, illustrated. Blue muslin, $2.25 net. New York: William Wood & Co. 1898.

Wounds in War; the Mechanism of Their Production and Their Treatment. By Surgeon-Colonel W. F. Stevenson, Professor of Military Surgery, Army Medical School, Netley, England. One volume of 588 pages, 8vo, profusely illustrated. Muslin, $4.00 net. New York: William Wood & Co. 1898.

A Treatise on the Principles and Practice of Gynecology. By E. C. Dudley, A. M., M. D., Professor of Gynecology in the Chicago Medical College, Chicago. In one octavo volume of 632 pages, with 422 engravings, of which 47 are in colors, and two colored plates. Cloth, $5.00 net; leather, $6.00 net. New York and Philadelphia: Lea Brothers & Co. 1898.

Tropical Diseases. A Manual of the Diseases of Warm Climates. By Patrick Manson, M. D., LL. D. (Aberd.), London. One volume of 625 pages, 12mo, illustrated by 88 wood engravings and two colored plates. Muslin, $3.50 net. New York: William Wood & Co. 1898.

A Manual of General Pathology for Students and Practitioners. By Walter Sydney Lazarus-Barlow, M. D., M. R. C. P., late Demonstrator of Pathology in the University of Cambridge, etc. Octavo, pp. XI.-795. Price, $5.00, net. Philadelphia; P. Blakiston's Son & Co. 1898.

Text-Book of Medical Jurisprudence and Toxicology. By John J. Reese, M. D., late Professor of the Principles and Practice of Medical Jurisprudence in the University of Pennsylvania. Fifth edition, revised and enlarged by Henry Leffmann, M. D., Pathological Chemist, Jefferson Medical College Hospital, Chemist State Board of Health, Professor of Chemistry, Woman's Medical College of Pennsylvania, etc. 640 pages. Cloth, $3.00; sheep, $3.50. Philadelphia, P. Blakiston's Son & Co. 1898.

Manual of the Diseases of Children. By John Madison Taylor, A. M., M. D., Professor of Diseases of Children, Philadelphia Polyclinic; Assistant Physician to the Children's Hospital and to the Orthopedic Hospital; Consulting Physician to the Elwyn and the Vineland Training Schools for Feeble-Minded Children; Neurologist to the Howard Hospital, etc.; and William H. Wells, M. D., Adjunct Professor of Obstetrics and Diseases of Infancy in the Philadelphia Polyclinic; late Assistant Demonstrator of Clinical Obstetrics and Diseases of Infancy in Jefferson Medical College. With 60 illustrations. 650 pages. Price, $4.00 net. Philadelphia: P. Blakiston's Son & Co. 1898.

Handbook of Materia Medica for Trained Nurses, including sections on Therapeutics and Toxicology, and a Glossary of Terms, with Dose and Use of Each Drug. By John E. Groff, Ph. G., Apothecary in the Rhode Island Hospital, Providence. 12mo, pp. 243. Price, $1.25 net. Philadelphia: P. Blakiston's Son & Co. 1898.

